# Pathology of the Mouth

**Published:** 1860-05

**Authors:** 


### DENTAL REGISTER OF THE WEST.

VOL. XIII.] MAY, 1860. [No. 11.

### Original Essays and Communications.

### PATHOLOGY OF THE MOUTH.

#### ACUTE CATARRH OF THE MOUTH.

THE phenomena of catarrh in the mouth are plainly exemplified in the well known symptoms of what is commonly called "a cold in the head," or in influenza. Catarrh is by no means confined to any locality, it may be manifested in any portion of the mucous membrane, but is more disposed to attack such portions of the mucous tissue as are exposed to atmospheric or other injurious influences. Perhaps the lining of the respiratory organs are more liable to this form of disease; next, the lining of the alimentary canal; then, the lining of the genital organs; and lastly, that of the urinary organs.

But frequently only parts of the mucous membrane of those systems are affected with catarrh, or different parts of it are affected with the disease in different degrees of intensity. The mouth, being connected with both the respiratory and the digestive organs, is liable to become diseased in connection with catarrh of either the lungs and the stomach, and hence catarrh of the mouth is a somewhat common form of disease.

The phenomena of a catarrh, and the conditions that produce those phenomena, wherever the disease may be located, are :—

*Firstly.* Congestion of the vessels of the mucous membrane and the immediately subjacent structures, identical in character with the congestion that is present in phlegmonous and fibrinous exsudative inflammation but without the throbbing and pain which occurs in congestion without those exsudations. For, as the exsudation in catarrhal inflammation is mucus, and the mucus formed is thrown *out* of the membrane and organ affected, it can not cause that obstruction to the flow of blood through the arteries that fibrinous exsudation does, and which is the cause of the throbbing, heat, and pain, felt in some forms of inflammation.

*Secondly.* The secretion of the part affected is first and briefly diminished; but quite soon it is greatly increased. In fibrinous inflammatory exsudation, as the amount of fibrine exsuded into the organ is increased the excretion of mucus is diminished.

*Thirdly.* Not only is there an alteration in the quantity but also quality of the fluid exsuded, and that change in quality is quite different in different localities. In nasal catarrh, in place of the usual pituital mucus there is frequently thrown out a watery, saltish, serous fluid that excoriates the skin over which it flows, dissolving and washing away the epithelium and epidermis and leaving the dermis exposed to the action of this fluid and to the oxygen of the atmosphere. At another time a thickened mucus will be poured out in considerable quantities, with its color altered from the pus globules and the vast number of detached epithelial scales it contains together with shreds of detached epithelium, and occasional flakes of fibrine. In some forms of febrile gastric catarrh there is a formation of imperfect epithelial scales in very large quantities. This condition when in the stomach is indicated by a *yellow* coat upon the tongue, a bitter taste in the mouth, and an unusual adhesiveness of the coating to the tongue.

There are three prominent forms of catarrh, each perhaps shading towards, and perhaps running into the other. They are those here indicated; or the *Serous*, the *Muco-purulent* and the *Epithelial* forms of the disease.

*Serous* catarrh is not often observed in the mouth, but at times the amount of *Mucus*, or a *Muco-purulent* fluid poured out into the mouth is very great. At such times the mouth appears to suffer from a derangement very similar to that of common inflammation. The tongue is white, not very heavily coated, at first moist or even wet, but toward the later stage of the disease frequently quite clean. The mucus poured into the mouth is swallowed, and is, together with the gastric mucus, often vomited in large quantities, or is passed from the bowels in thick stringy lumps. As the febrile symptoms that accompany this form of catarrh are apt to be remittent, this disease has been known as "Gastric Remittent Fever."

*Epithelial* catarrh of the mouth appears always to be connected with a similar catarrh of the stomach. It may be distinguished by the thick, heavy, yellow coat on the tongue, the bitter taste of the mouth, and the tenacious adhesiveness of the coating. Also by the stomach being incapable of digesting food, its being irritable, and quite apt to vomit its undigested contents. When there has been considerable retching, produced by the disease or by a nauseating emetic, large quantities of bile will be drawn into the stomach, which may be vomited along with the ropy tenacious mucus excreted by the stomach and swallowed into it from the mouth. If the bile is not vomited up it may pass off in considerable quantities by stool. This condition of the system has been expressed by saying the patient had a "Bilious attack."

The more common *cause* of these forms of buccal and gastric catarrhs is,—an *arrest* or partial arrest of the usual excretion through the skin. The perspiration is diminished or ceases entirely, the epidermis is not as freely shed and renewed as is necessary for health, the head becomes dry, and the skin on the hands is liable to crack. We know that those who labor under an abnormal excretion from the bowels, as in chronic diarrhœa, and from profuse, long-continued leucorrhœa, or from the profuse expectoration of consumption, have transparent skins and clean scalps; and we are justified in inferring that a suppression of the natural dermic secretion is often if not always the cause of buccal and gastric, as well as pulmonic and intestinal catarrh.

*Fourthly.* In catarrh, the nerves of the sub-mucous tissues are affected so as to produce the prickling sensation in the nose, the cough in pulmonary catarrh, the vomiting when the stomach is the seat of the disease, and the diarrhœa when the intestines are the organs affected.

*Fifthly.* There is a marked periodicity observable in all forms of catarrh wherever located. The febrile and catarrhal symptoms are less in the morning, and always grow worse towards evening. The disease, like the exenthemata when uncomplicated, ends within a few days.

*Treatment.*—The first thing to be attended to in all forms of catarrh, is to insure a free exit to the mucus from the system. From the mouth it is usually swallowed into the stomach. In the lungs if allowed to accumulate, or checked in its flow by the use of opiates, the patient certainly suffers. In the stomach and bowels, the mucus provokes vomiting or diarrhœa, and it is never well to arrest these entirely so long as the inordinate secretion continues. It may even be necessary to give emetics, but more particularly mild cathartics, to aid in expelling any accumulation of mucus. In epidemic catarrhs, mild warm purgatives with sudorigentic agents, are all the medicines physicians are called upon to administer.

Even when the patient is quite debilitated, mild but efficient measures should be taken to dislodge and evacuate any considerable accumulation of mucus, and if cautiously managed, the patient will feel stronger for having been relieved of the load that was weighing him down. When the stomach is thus embarrassed, it can not absorb food or medicine. But when it has been emptied of its thick layer of tenacious secretions, both medicine and food can be taken with benefit.

As an emetic in this disease, the common table salt, to induce free osmosis of the watery position of the blood into the stomach and detach the adherent mucus, will be found very beneficial. Vinegar in proper quantities is found to be an admirable solvent for the mucus, and as an astringent it checks its flow. An infusion of hydrastis Canadensis root, to be taken whenever the tongue or stomach have been cleansed so as to allow it to come directly in contact with the mucous coat, is a very superior article.

The bowels should be moved with the mildest and most gentle cathartics, mixed with a little bicarbonate of potassa, or with the chlorate of the same alkali.

In all forms of catarrh there is an unusual amount of excretion of the fluids of the body, and plenty of water in some form must be given to supply the waste. The thirst that is a usual accompaniment of catarrh is a natural instinctive one, and should be gratified.

*Diet.*—As there is a diminution of the digestive power, as well as an increased demand for fluids, liquid foods are found to be far preferable to solid ones. Solid meats should not be given to patients suffering from catarrh, but animal food in the fluid forms mentioned while treating of *Arrested Secretion*, will always be found beneficial. Especially should the food be suspended in considerable water in the epithelial form of the disease.

### CHRONIC CATARRH OF THE MOUTH.

This form of disease, especially when the mucous membrane of the fauces, throat, and stomach are involved, in addition to that of the buccal cavity, is often known as "mucus flux." Although closely allied in nature to acute catarrh, it does not appear to be the result of the acute form of the disease.

In chronic catarrh of the mouth and stomach, the tongue is smooth, glazy, and wet, with a quantity of white epithelium spread over the tongue and the lining of the cheeks.

Although this coating of epithelium is never very thick it is sometimes remarkably white.

Chronic catarrh does not, like the acute disease, tend to a speedy termination; but in spite of treatment, it at times will progress from bad to worse; or if apparently improving for a time, will grow worse again, and perhaps linger for years a constant torment to the sufferer.

Not only is the mucus increased in quantity in chronic catarrh; but as shown when speaking of the acute disease, it is also changed in quality, becoming tough and more than naturally adherent to the parts to which it is attached. It hence acts as a foreign substance, enveloping the food, impeding the flow of saliva and the gastric juice, and covers over the lymphatics and veins as well as the mucous membrane and thus hinders the absorption of food and drinks. As the food is not properly acted upon by the saliva and gastric juice, and is not readily absorbed, it undergoes fermentation and putrefaction in the stomach, and thus generates morbid acids and morbid gases, and causing uneasiness, distress, and not unfrequently severe pain in the stomach and bowels.

As the excessive vitiated mucus, and undigested, fermenting, putrifying food descends into the intestines, it causes constipation, griping, colic, and is finally evacuated in hard lumps covered with slime.

Mucus as has been shown, and especially the secretion of the mouth, is alkaline; and as it passes into the stomach in large quantities it neutralizes the acid natural in the gastric juice and renders that fluid incapable of digesting solid animal food. It also, by preventing the fluid being digested in, and absorbed from, the stomach, favors the formation of the acids of fermentation in the bowels.

As the stomach contains more mucus than usual and hence its contents are rendered alkaline, the alkalinity of the blood may be no greater, and perhaps be even less than that of the contents of the stomach, and thus the osmotic circulation may be outward into the stomach instead of into the system, and in this way catarrh of the mouth and stomach may cause a serous diarrhœa.

When there is an arrest of the secretion of gastric juice, as here indicated, and albumenoid food is eaten, the albumen is not digested, and passing into the intestines, sulphureted hydrogen gas is formed giving the very offensive odor to the gaseous eructations and the gas passed out of the bowels; and also, if it is absorbed into the system, poisoning the fluids, depressing the vital energies, and finally escaping through the lungs and skin and rendering the breath and cutaneous exhalations quite offensive.

Starchy and saccharine foods can be digested in small quantities: but if taken in larger amounts than can be digested, and especially if mixed with animal food, the mass quickly undergoes decomposition and causes distress in the various ways here described. Or it may quite soon be vomited from the stomach along with the mucus.

If much mucus is vomited from the stomach after a meal, it is always alkaline; but if the food has been in that viscus long enough to become fermented, the liquid will be quite acid, causing acid eructations so acid as to set the teeth on edge and fill the room with a powerful penetrating odor of acetic acid. At times there will be observed lumps of alkaline mucus floating in the acid fluid vomited up.

The *Treatment* already detailed as applicable to acute catarrh of the mouth, will be found useful in the chronic form of the disease. Chlorate of potassa, hydrastis Canadensis, asarum, and other medicines found to be useful in inflammation of the mucous membrane, may be applied in the form of gargles and lotions. Sometimes the vapors of medicine seem to be more efficacious than the infusions or solutions.

The peculiar derangements pointed out as the results of the disease, will each demand its own peculiar treatment, which may be adopted in conjunction with that directed to the original malady.

